# The Mitochondrial Genome Assembly of Fennel (*Foeniculum vulgare*) Reveals Two Different *atp6* Gene Sequences in Cytoplasmic Male Sterile Accessions

**DOI:** 10.3390/ijms21134664

**Published:** 2020-06-30

**Authors:** Fabio Palumbo, Nicola Vitulo, Alessandro Vannozzi, Gabriele Magon, Gianni Barcaccia

**Affiliations:** 1 Department of Agronomy Food Natural Resources Animals and Environment (DAFNAE), University of Padova, Campus of Agripolis, Viale dell’Università 16, 35020 Legnaro, PD, Italy; alessandro.vannozzi@unipd.it (A.V.); gabriele.magon@phd.unipd.it (G.M.); gianni.barcaccia@unipd.it (G.B.); 2 Department of Biotechnology, University of Verona, Strada Le Grazie 15, 37134 Verona, Italy; nicola.vitulo@univr.it

**Keywords:** male sterility, ATP synthase, mtDNA, organelle assembly, energy deficiency model, F1 hybrid breeding, pollen, marker-assisted selection

## Abstract

Cytoplasmic male sterility (CMS) has always aroused interest among researchers and breeders, being a valuable resource widely exploited not only to breed F1 hybrid varieties but also to investigate genes that control stamen and pollen development. With the aim of identifying candidate genes for CMS in fennel, we adopted an effective strategy relying on the comparison between mitochondrial genomes (mtDNA) of both fertile and sterile genotypes. mtDNA raw reads derived from a CMS genotype were assembled in a single molecule (296,483 bp), while a draft mtDNA assembly (166,124 nucleotides, 94 contigs) was performed using male fertile sample (MF) sequences. From their annotation and alignment, two *atp6*-like sequences were identified. *atp6*^−^, the putative mutant copy with a 300 bp truncation at the 5’-end, was found only in the mtDNA of CMS samples, while the wild type copy (*atp6*^+^) was detected only in the MF mtDNA. Further analyses (i.e., reads mapping and Sanger sequencing), revealed an *atp6^+^* copy also in CMS samples, probably in the nuclear DNA. However, qPCRs performed on different tissues proved that, despite its availability, *atp6^+^* is expressed only in MF samples, while *apt6^−^* mRNA was always detected in CMS individuals. In the light of these findings, the energy deficiency model could explain the pollen deficiency observed in male sterile flower. *atp6*^−^ could represent a gene whose mRNA is translated into a not-fully functional protein leading to suboptimal ATP production that guarantees essential cellular processes but not a high energy demand process such as pollen development. Our study provides novel insights into the fennel mtDNA genome and its *atp6* genes, and paves the way for further studies aimed at understanding their functional roles in the determination of male sterility.

## 1. Introduction

Plant male sterility (MS), is generally defined as the inability of plants to produce functional pollen, dehiscent anthers, or viable male gametes [1]. According to their mode of inheritance, two types of MS are recognized: Cytoplasmic male sterility (CMS), which is caused by mitochondrial genes coupled with fertility restoring nuclear genes, and nuclear or genic male sterility (NMS or GMS), determined exclusively by nuclear genes [2].

Both types of MS are currently exploited for breeding purposes and hybrid constitution and the worldwide crop production has tremendously benefited from their utilization. It is estimated that hybrid vigor can offer, on average, from 20% to over 50% increases [3] and this would explain why hybrid seeds market, valued at $52 billion in 2016, is expected to reach at $99 billion by 2023, with a Compound Annual Growth Rate (CAGR) of 9.7% [4].

CMS is more widely used than GMS because this maternally inherited trait can be applied, preserved and restored more efficiently [5,6]. In fact, a CMS-based hybrid seed technology only requires a CMS line, a sterility maintainer line, and a fertility restorer line [7,8]. Moreover, in addition to its wide application in hybrid production, CMS provides important resources to investigate stamen and pollen development and to study the crosstalk between nuclear and mitochondrial genomes [9]. Most of the CMS genes reviewed by Chen and Liu [1] belonged to the mitochondrial electron transfer chain (mtETC) or to the ATP synthase complex and, among them, it is worth mentioning *cox1* [9,10], *atp8* [11], and *atp6* [5,12,13]. In other cases, species-specific mitochondrial open reading frames (ORFs) encoding for transmembrane proteins were found to be responsible for CMS [14,15]. According to the National Center for Biotechnology Information (NCBI) [16] CMS-related genes have been identified and deposited for at least 76 plant species, including cereals such as bread wheat (*Triticum aestivum*) [17], rice (*Oryza sativa*) [18], and maize (*Zea mays*) [19], and crops like stem mustard (*Brassica juncea*) [20,21], onion (*Allium cepa*) [9], pepper (*Capsicum annuum*) [5], radish (*Raphanus sativus*) [22], sunflower (*Helianthus annuus)* [23], and carrot (*Daucus carota*) [11]. In fennel (*Foeniculum vulgare*), the presence of a naturally occurring NMS system has never been reported while several spontaneous CMS mutants have been discovered and exploited by breeders since the late 1960s [24,25]. These mutants are widely used for the development of F1 hybrids [2] that, today, represent the 95% of the cultivated fennel varieties [26]. Strikingly, despite its economic relevance (more than 1 million tons produced in 2018, with a gross production value of $6.5 billion [27]), the genetic mechanism underlying the cytoplasmic male sterility in fennel is far from being understood and no candidate gene has been identified yet. What is more, the only biological data currently available for this species in public databases (e.g., GenBank) are the plastome [28], an early genome draft [2] and a leaf transcriptome [29].

It is now well understood that one of the most effective strategies to identify CMS candidate genes relies on the comparison between mitochondrial genomes of both fertile and sterile genotypes, to search for differences in terms of sequences or structure organization [30,31]. In the last few years the identification of such genes has been greatly facilitated by the advent of Next Generation Sequencing (NGS): To date 311 plant mitochondrial genomes are available in the NCBI organelle genome database [32] and this number is constantly increasing thanks to the ongoing evolution of sequencing technology and the decrease in sequencing costs [33].

Following this approach, in the present study we performed a fennel genome mitochondrial assembly based on data collected from public repositories and we then looked for nucleotide variants comparing sequences from both fertile and sterile lines.

## 2. Results

### 2.1. Mitochondrial Genome Sequence and Annotation

**Figure 1:**
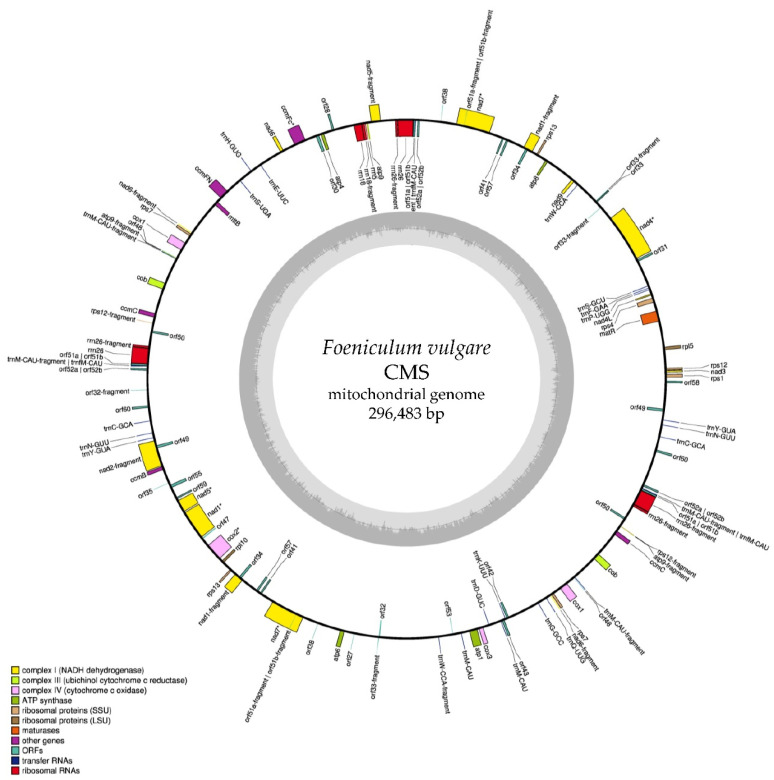
Annotation of *Foeniculum vulgare* mitochondrial genome assembled from a cytoplasmic male sterile (CMS) accession. The annotation was created with GeSeq [34] and visualized with OGDRAW. Genes marked with an asterisk (*) contain introns.

486,047,668 reads Illumina HiSeq 2500 (150 bp PE, Illumina, Inc., San Diego, CA, USA) derived from the Whole Genome Sequencing (WGS) of a cytoplasmic male sterile (CMS) accession (SRX7730623) of *Foeniculum vulgare*, were assembled in a single mtDNA molecule of 296,483 bp with a GC contents of 45.9% (Supplementary File 1). Mapping back the reads to validate the mtDNA assembly, the proportion of properly aligned reads was 0.3% with an average coverage depth of 758×. From the annotation step performed through GeSeq [34] 24 mtDNA genes, 26 ORFs, 25 tRNAs, and 9 rRNAs were identified (Figure 1, Supplementary File 2).

In parallel, 17,377,226 reads Ion GeneStudio S5 System (400 bp SE, Thermo Scientific, Pittsburgh, PA, USA) were retrieved from the WGS of a male fertile (MF) fennel accession (SRX2770225) and assembled in 94 contigs (Supplementary File 3), ranging from 168 bp to 10,174 bp (total length 166,124 bp, N50 = 4702, L50 = 12) and a GC contents of 46.3%. The percentage of raw reads that were correctly mapped back to the MF mtDNA contigs was 0.4%, with an average coverage depth of 151×. GeSeq [34] allowed for the identification and annotation of 24 mtDNA genes, 24 ORFs, 19 tRNAs, and 9 rRNAs were identified (Supplementary File 4).

From a comparison among the CMS and MF mitochondrial genomes, a one-to-one correspondence was found for three ribosomal protein large subunits (*rpl2*, *rpl5*, *rpl10*), six ribosomal protein small subunits (*rps1*, *rps3*, *rps4*, *rps7*, *rps12*, *rps13*); nine nadh dehydrogenase subunits (*nad1*, *nad2*, *nad3*, *nad4*, *nad4L*, *nad5*, *nad6*, *nad7*, and *nad9*, complex I), three cytochrome oxidase subunits (*cox1*, *cox2*, and *cox3*, complex IV), cytochrome b (*cob*, complex III), five atp synthase subunits (*atp1*, *atp4*, *atp6*, *atp8* and *atp9*, complex V), four cytochrome C synthesis related proteins (*ccmB*, *ccmC*, *ccmFc* and *ccmFn*), *matR,* and *mttB*.

### 2.2. SNP and InDel Detection

In order to identify sequence variations between the MF and CMS accessions, SNPs and InDels were searched aligning the mtDNA MF contigs against the CMS mtDNA. As shown in Table 1, 63 variants, namely 36 SNPs and 27 InDels, were detected. 

**Figure 2:**
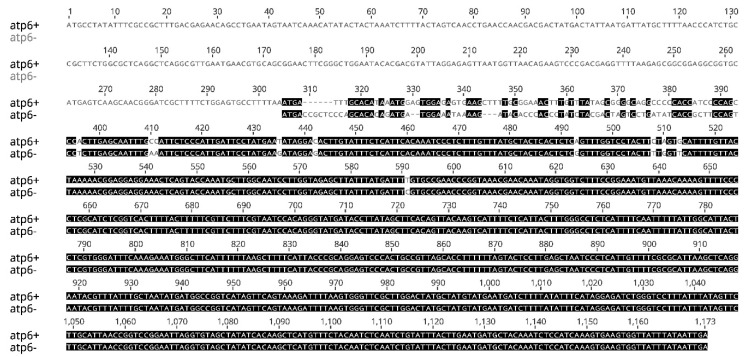
Nucleotide acid alignment of *atp6* genes sequences retrieved from the mtDNA of male fertile (MF, *atp6^+^*) and cytoplasmic male sterile (CMS, *atp6^−^*) accessions of *Foeniculum vulgare*.

Among the sequence variations, 22 occurred in extra genic regions, 6 within intron regions and, as a main finding, *atp6* and *rps3* were the only two CDS regions showing nucleotide variants between fertile and sterile samples (Table 1, in bold). As regards the *rps3* gene (1659 bp), all the 3 SNPs resulted non-synonymous (Figure S1), while for *atp6*, 31 variants were responsible for 28 amino acid mismatches (Figure S2). More interestingly, the first 300 bp of the MF *atp6* copy (from now on defined as *atp6^+^*, 1167 bp) were missing in the *atp6* copy (henceforth *atp6^−^*, 864 bp) found in male sterile mtDNA (Figure 2).

### 2.3. Selection of Candidate Gene Controlling Cytoplasmic Male Sterility

**Figure 3:**
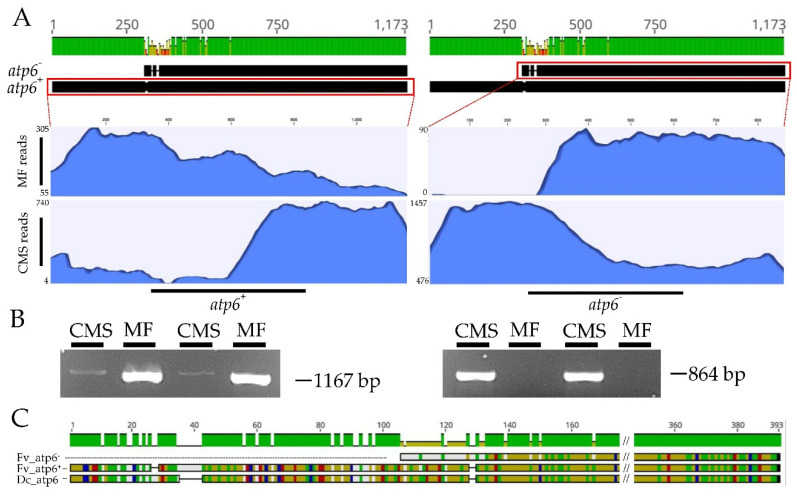
(**A**) Nucleotide alignment between the two *atp6* copies retrieved within the mitochondrial genomes of *Foeniculum vulgare. atp6*^+^ indicates the wild type gene (1167 bp) found in the male fertile (MF) mtDNA while *atp6*^−^ is an *atp6*-like sequence lacking the first 300 bp (864 bp) retrieved in the cytoplasmic male sterile (CMS) mtDNA. Raw sequencing reads from MF and CMS accessions were mapped to *atp6^+^* and *atp6^−^*. (**B**) PCR profiles generated in two CMS commercial lines and their MF maintainers with specific primers for *atp6*^+^(left) and *atp6^−^* (right). (**C**) Protein alignment of *atp6*^+^, *atp6*^−^, and the *atp6* protein from *Daucus carota* (Dc_atp6).

Following the results obtained for *atp6*, raw sequencing reads from both MF and CMS GenBank accessions were mapped back to *atp6^+^* and *atp6^−^*. As reported in Figure 3, panel A, MF reads were successfully mapped only to the *atp6^+^* copy, while CMS reads aligned both against *atp6^+^* and *atp6^−^*. However, the alignment coverage of these latter reads against the *atp6^+^* copy was considerably lower than the coverage exhibited against the *atp6^−^* (Figure 3, panel A).

The results obtained from the reads mapping were further confirmed by Sanger sequencing two different CMS lines and their MF maintainers by means of two different gene-specific primer sets. As shown in Figure 3, panel B, male fertile samples exhibited PCR amplicons only for *atp6^+^*, while the CMS accessions were successfully amplified at both loci. Although *atp6^+^* primers binding sites were found conserved in both CMS and MF accessions, the intensity of the band for *atp6^+^* was very low for male sterile individuals (Figure 3B, the original picture of the agarose gel is available as Figure S3). The same result was also confirmed in a further amplification experiment conducted on four additional different commercial CMS accessions and their MF maintainers (Figure S4). 

Finally, from the alignment of the resulting proteins of *atp6^+^* (388 amino acids) and *atp6^−^* (287 amino acids) with atp6 of *Daucus carota* (Dc_atp6, 383 amino acids), the percentage of pairwise identical residues was 92.3% between *atp6^+^* and Dc_atp6 and 90.2% between *atp6^−^* and Dc_atp6 (Figure 3, panel C). This finding, along with the comparable sequence length of *atp6^+^* and Dc_atp6 (i.e., 388 vs. 383 amino acids), led to the hypothesis that *atp6^+^* represents the wt allele.

### 2.4. Expression Analyses of atp6^+^, atp6^−^, atp1, and atp9

**Figure 4:**
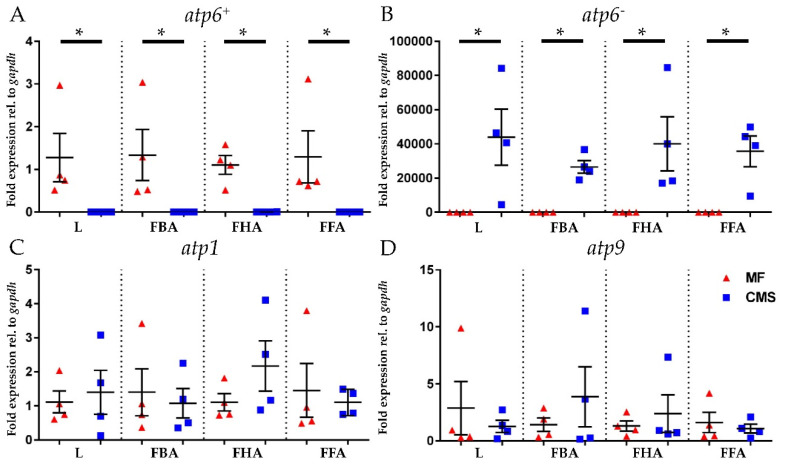
Evaluation of *atp6^+^* (**A**), *atp6^−^* (**B**), *atp1* (**C**), and *atp9* (**D**) mRNA expression by qPCR analysis. Expression analysis were conducted on male fertile (MF, n = 4, red triangles) and male sterile samples (CMS, n = 4, blue squares). Four different tissues were evaluated for each gene: leaf (L), flower before anthesis (0% flowering, FBA), flower in half anthesis (50% flowering, FHA) and flower in full anthesis (100% flowering, FFA). Ct values were normalized for *gapdh*. Statistical significance was evaluated with unpaired Student’s *t*-test (* *p* < 0.05). All data are shown as mean ± SD.

The expression patterns of four genes, *atp6^+^*, *atp6^−^ atp1,* and *atp9* were successfully analyzed in leaves and flowers at three different developmental stages (0% flowering, 50% flowering and 100% flowering) of two CMS commercial lines and their MF maintainer (Figure 4). *atp6^+^* and *atp6^−^* were found to be constantly and exclusively expressed only in MF or CMS, respectively (Figure 4, panels A and B). Vice versa, *atp1* and *atp9*, used here as positive controls, were constantly expressed in all the tissues considered without significant differences between CMS and MF samples (Figure 4, panels C and D).

## 3. Discussion

In the last five years, the knowledge about extranuclear genomes of the Apiaceae family, whose aromatic plants are extensively used for several purposes, including food products (e.g., celery and fennel), beverages (e.g., carrot), flavoring components (e.g., cumin), cosmetics (e.g., tōki), and pharmaceuticals (e.g., carrot and fennel) [35,36], has considerably advanced thanks to the advent of NGS technologies. Several studies have greatly contributed not only to uncover the sequence, but also to comprehend the structure, expression, and variation of these organelles [37,38,39]. However, it must be acknowledged that in the face of 42 chloroplast (cp) genomes deposited in NCBI from as many Apiaceae species, only two mitochondrial (mt) genomes are available (i.e., *Daucus carota* and *Bupleurum falcatum*). A similar discrepancy is also observed at a wider level (i.e., plant level), being the number of plastid genomes (3.720) accessible in NCBI ten times larger than that of mitochondrial assemblies (311). This numerical disproportion can be attributed to several factors. First, in contrast to the cpDNA genome, mtDNA is more often characterized by heteroplasmy phenomena (i.e., co-existence of additional mitochondrial genome types called mitotypes) [40] and by a variety of different arrangements and configurations (circular, linear or branched molecules) [41,42,43] that can strongly affect their correct and exhaustive assembly. Moreover, when compared to cpDNA genomes, mtDNA has proven to be bigger and to contain more repeat regions as well as significant amounts of nuclear and chloroplast genomic sequences [44]. All these peculiarities, along with technical issues linked to the amount and the type of available reads (short vs. long), may limit the accurate assembly of this organelle genome. These observations would explain the different findings achieved in this study for the mtDNA assembly of male sterile and male fertile accessions of *Foeniculum vulgare*. We successfully managed to assembly the CMS mtDNA in a single molecule while the MF mtDNA was assembled only to a contig level. The main reason behind this discrepancy lies on the huge gap in terms of raw sequences available for the two contrasting phenotypes, being the MF reads accessible in GenBank less than a twentieth of the CMS ones. In fact, the poor coverage characterizing MF reads has complicated the assembly of extra genic regions and repetitive structures leading to the translation into gaps and incomplete results. On the contrary, the assembly of genic regions were comparable between the two mtDNAs and, in both cases, we were able to reconstruct the full sequences of 33 genes (excluding ORFs and tRNAs). This number is in agreement with what reported by Kubo and Newton [45], and Morley and Nielsen [46]: Excluding tRNAs and all the species-specific ORFs, the number of mitochondrial genes usually ranges from 27 (*Beta vulgaris*) to 39 (*Zea Mays*). 

Based on the most complete assembly (the CMS one), the estimated size of the fennel mtDNA assembled in this study is 296,483 bp, 1.94 greater than the fennel plastome (153 kb) assembled by Peery et al. [28]. This is in line with what observed for *Daucus carota* (from the same family, Apiaceae) whose mtDNA is 281,132 bp in length and the ratio mtDNA/cpDNA is 1.80.

The SNP calling analysis, performed mapping the MF contigs to the CMS mtDNA, allowed us to detect 42 polymorphic loci able to discriminate the two antagonist phenotypes (i.e., MF and CMS plant genotypes) and, most importantly, to reveal the presence of two different *atp6* copies. *atp6^+^*, considered the wild type copy based on the highest similarity with *atp6* in *Daucus carota* was detected only in the male fertile mtDNA, *atp6^−^*, the putative mutant copy with a 300 bp truncation at the 5’-end, was available only in the mtDNA of male sterile samples. As already observed for *Rhazya stricta* [47] and *Arabidopsis thaliana* [48], *atp6*, compared to other mt genes, is often characterized by extended repeat sequences located at the upstream region suggesting that this gene is more likely involved in genomic recombination events. Despite the data available for the MF mtDNA are not sufficient to demonstrate the presence of repeated regions in the 5’ region of *atp6^+^*, it is reasonable to assume that the truncation of 300 bp in *atp6^−^* is attributable to a mitochondrial rearrangement.

Following the validation analyses (i.e., mapping back the sequencing reads to both *atp6* copies and performing PCR based on copies-specific primers) two main findings were achieved. First, *atp6^−^* was confirmed to be present only in the CMS samples and, thus, the F2/R2 primer couple (namely the one used to specifically amplify the *atp6^−^* copy) can be directly used in a molecular assay suitable for breeding purposes and for selecting only CMS samples. Second, *atp6^+^* was detected also in CMS samples. However, the intensity of the *atp6^+^* PCR band and the reads coverage was lower when compared to the mutant (*atp6^−^*) counterpart (Figure 3A,B and Figures S3 and S4), suggesting that *atp6^+^* is present as single copy in the nuclear genome, while *atp6^−^* is present in the mitochondrial genome, as supported by the CMS mtDNA assembly, the higher sequencing coverage and greater band intensity. A possible transfer of the *atp6* gene to the nuclear genome would not be exclusive to *Foeniculum vulgare:* Genes duplication and/or relocation from the mitochondrion to the nucleus is a recurrent and consistent feature of eukaryotic genome evolution [49] and this phenomenon has already been described both in plants [50] and animals [51]. This was also confirmed in *Daucus carota* from an *ex-post* analysis that we performed aligning the mitochondrial *atp6* gene (NC_017855.1:6101-7252) to the entire genome: An almost identical copy of this gene was found also within the chromosome 7 (NC_030387.1:11491922-11492723, E-value = 0.0, % identity = 99.50). It is also reasonable to think that a nuclear copy of *atp6* is also present in MF genotypes, but, unfortunately, this hypothesis cannot be verified because the only whole genome sequencing available for fennel comes from a CMS individual [2].

**Figure 5:**
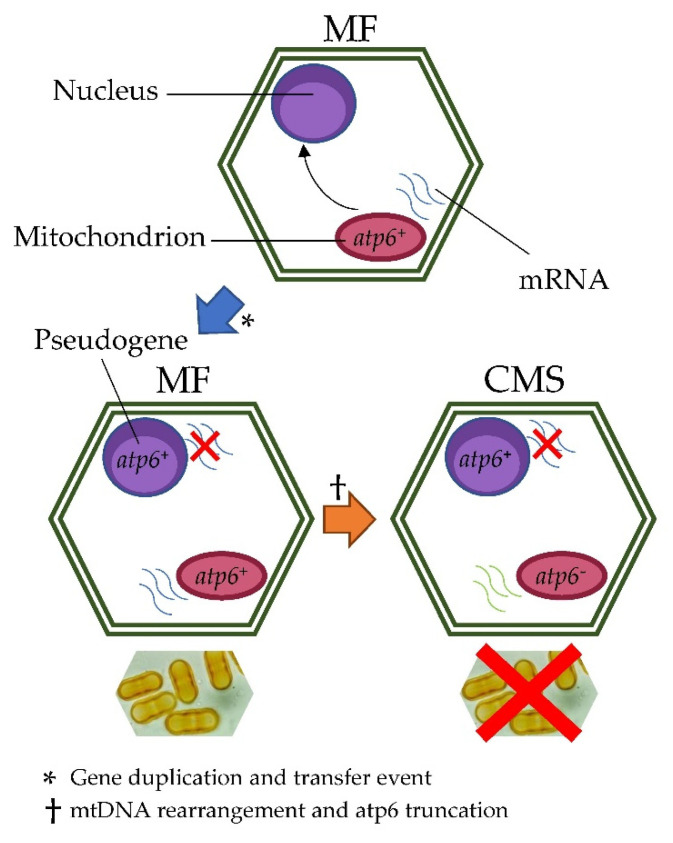
Involvement of *atp6* in cytoplasmic male sterility in fennel. The *atp6^+^* gene duplication and transfer from the mtDNA to the nuclear genome is a recurrent and consistent feature of eukaryotic genome evolution that seems to have characterized also the evolutionary path of *Foeniculum vulgare*. Subsequently, a mitochondrial rearrangement event produced a truncation of 300 bp in the mtDNA *atp6* copy (*atp6^−^*) leading to a cytoplasmic male sterility phenotype. Expression analyses revealed that (i) *atp6^+^* gene is constantly expressed in male fertile plants (MF), where it is available both in the mtDNA and, probably, in the nuclear DNA (ii) *atp6^+^* gene is never expressed in male sterile plants, where is available only in the nuclear DNA (probably as pseudogene) (iii) *atp6^−^* is constantly expressed in male sterile plants (MF), where is available in the mtDNA.

The transcription analyses added another piece to this increasingly complex puzzle. While other ATP synthase subunits (i.e., *atp1* and *atp9*) were constantly and equally expressed (i.e., no significant expression difference observed between CMS and MF), *atp6^+^* was ubiquitously expressed in all the tissues of the MF samples and never detected in any CMS tissues. Thus, it can be postulated that the nuclear *atp6^+^* copy is probably a pseudogene. Moreover, despite the 300 bp truncation at the 5’-end, *atp6^−^* mRNA was found to be constantly available in all the tissues analyzed for the CMS. The main findings are summarized in Figure 5.

It is well known that *atp6* is a key component of the transmembrane F_o_ portion of the ATP synthase and it is frequently associated with CMS phenomena both in monocotyledon species such as rice [52], maize [53], and dicotyledon species such as pepper [5], sugar beet [13], brown mustard [12], carrot [39], kenaf [54], and sunflower [55].

Our results fit with the “energy deficiency model” according to which, the partial or total loss of function of one or more subunits belonging to the mtETC (complexes I-IV) or to the ATP synthase (complex V), can determine mitochondrial deficiencies [1]. This is supported by several evidences available in the scientific literature. In CMS sugar beet a truncated copy of *cox2* (a subunit of complex IV) showed a decreased activity when compared to the wt copy and proved a molecular link between mtETC defect and male sterility [56]. In *pol* CMS of *Brassica napus* the mutant *atp6* protein is thought to diminish the functionality of the complex V [57] while in CMS sunflower a chimeric protein orf522 seems to reduce the ATPase activity [58]. In *Foeniculum vulgare*, based on our findings, we assumed that *atp6^−^*, being the only gene expressed in CMS fennel plants, must be also translated into protein. It can be assumed that *atp6^−^* proteins may affect ATP production by forming less efficient ATPase complexes that guarantee essential cellular processes (and thus the plant growth) but not a high energy demand process such as pollen development. Further analyses of post-translation events are necessary to determine the role of *atp6^−^* on the male-sterility induction.

In conclusion, our study provides useful findings and novel insights into the fennel mtDNA genome and its *atp6* genes, and paves the way for functional studies in order to shed light on their roles in the determination of male sterility.

## 4. Materials and Methods 

### 4.1. Assembly of Mitochondrial DNA

Two SRA sequence datasets, namely SRX2770225, and SRX7730623, were downloaded from the National Center for Biotechnology Information (NCBI) GenBank, under the taxonomy ID: 48038 (*Foeniculum vulgare*). The first archive was obtained from a male fertile sample (hereafter named “MF reads”), whereas SRX7730623 reads derived from a cytoplasmic male sterile accession (hereafter named “CMS reads”). 

CMS reads were assembled using GetOrganelle software [59], a fast de novo genome organelle assembler. The program was run providing as seed the mitochondrial genome of *D. carota* (GenBank accession NC_017855.1), using the option -fast and setting the k-mers length used by SPAdes assembler [60] to 21, 65, and 105.

Since GetOrganelle is not optimized to run with Ion GeneStudio S5 sequences, an alternative approach was used instead to assemble the MF mitochondrial genome. At first, the reads set was aligned to the CMS mitochondrial genome using Bowtie2 program [61]. All the mapping reads were retrieved, and de novo assembled using SPAdes [60]. Spades was run setting the option –iontorrent and adjusting the k-mers length to 21,33,55,77,99 and 127.

Both genomes were finally annotated by means of GeSeq [34] using the mtDNA of *Daucus carota* (NC_017855.1) as a reference.

### 4.2. Variant Calling and Sanger DNA Sequencing Validation

The two new assembled genomes were compared to detect any variant. Contigs assembled from MF reads were mapped against the CMS mtDNA, using the “basic variant detection” tool (CLC Genomics Workbench 11.0.1, CLC Bio). 

Following the results obtained from the variant calling analysis, raw reads from both MF and CMS accessions were mapped against the *atp6* gene copy found within the mtDNA fertile (defined as *atp6^+^*) and the *atp6* gene copy found within the mtDNA sterile (named *atp6^−^*). Mapping was performed using the “map reads to contigs” tool of CLC Genomics Workbench 11.0.1 software (CLC Bio, Århus, Denmark) at default settings.

The two *atp6* gene copies were also validated through a Sanger sequencing. Geneious 11.1.4 software (http://www.geneious.com [62]) was used to design primers, for the specific amplification of both *atp6* copies. In details, for the analysis of *atp6^+^*, the following primers were used: F1: ATCGACCTGAACAACATATACGGA R1: GCTGGCGATTTCCGACAAGT while for *atp6^−^*, F2: TCACTGAGCACTGTCTG and R2: CCTAGAGTCTTTCGATACTATA were employed. PCR reactions were performed on genomic DNA purified (DNAeasy Plant Mini Kit, Qiagen, Valencia, CA, USA), from 4 samples: Two CMS individuals from as many commercial lines and two samples from their two isogenic MF maintainers. Reactions were set up in a 20 µL reaction volume, containing 1× Platinum^®^ Multiplex PCR Master Mix (Thermo Scientific, Pittsburgh, PA, USA), 0.5 µM of primers, 15 ng of genomic DNA and water up to volume A Veriti 96-Well Thermal Cycler (Applied Biosystems, Foster City, CA, USA) was programmed with the following conditions: initial denaturation at 95 °C for 5 min followed by 40 cycles at 95 °C for 30 s, 58 or 53 °C (*atp6^+^* or *atp6^−^*, respectively) for 30 s, 72 °C 90 s, and final extension of 10 min at 72 °C. Amplification was evaluated on a 1.5% (w/v) agarose gel stained with 1 x SYBR Safe^TM^ DNA Gel Stain (Life Technologies, Carlsbad, CA, USA). Amplicons were purified through ExoSap-IT (Applied Biosystems), sequenced (ABI 3730 DNA Analyzer, Applied Biosystem) using both forward and reverse gene specific primers, analyzed in Geneious 11.1.4 and deposited to GenBank (MT199323 and MT199324).

### 4.3. RNA Isolation and Quantitative Real-Time PCR

The same four lines used for DNA sequencing validation (i.e., two CMS commercial lines and their two isogenic MF maintainers) were used for expression analyses. From each line we collected (in two biological replicates) four tissues: leaf, flower before anthesis (0% flowering), flower in half anthesis (50% flowering) and flower in full anthesis (100% flowering). Samples were ground in liquid nitrogen, and total RNA was extracted using the Spectrum^TM^ Plant Total RNA Kit (Sigma-Aldrich, St. Louis, MO, USA) following the manufacturer’s instructions. RNA quality and quantity were checked by means of conventional electrophoresis and spectrophotometry using a NanoDrop-1000 (Thermo Scientific). cDNA was synthetized starting from 500 ng of RNA using the SuperScript^TM^ IV VILO Master Mix (Thermo Scientific) according to the manufacturer’s instructions. 

Five genes, namely *atp6^+^, atp6^−^, atp1,* and *atp9* and *gapdh* (this latter used as housekeeping gene) were selected and primers were designed using Geneious 11.1.4 (Table S1). All the qPCRs were performed on a QuantStudio 3 machine (Thermo Scientific) following the PowerUp SYBR Green Master Mix method (Applied Biosystems). Each reaction was carried out in a volume of 10 µL containing 5 µL of 2× SYBR Green, 0.5 µM of primers, 2 µL of 1:10-diluted cDNA and water up to volume. The run method set was as follows: initial denaturation 95 °C for 20 s, followed by 40 cycles of denaturation at 95 °C for 3 s and primer annealing, extension and gathering the fluorescence signal at 60 °C for 30 s. Subsequently, the melting curve analysis was achieved to verify the specificity of the primer with the following program: 95 °C/15 s, 60 °C/1 min, and 95 °C/15 s. The output data were analyzed according to Livak et al. [63]. The baseline and threshold cycles (Ct) were automatically determined by the software of the system and the three technical replicates taken for each biological replicate were averaged. The resulting mean Ct values were normalized as difference in Ct values (ΔCt) between the analyzed mRNA and *gapdh* reference gene. The ΔCt values of each sample were then normalized with respect to the ΔCt values of the MF samples (ΔΔCt). Finally, the variation was reported as fold change (2^−ΔΔCt^).

## Figures and Tables

**Table 1:** Variant analysis results. Variant location in CMS mtDNA, variant type (Ins = Insertion, Del = Deletion, SNV = Single Nucleotide variant, MNV = Multiple Nucleotide Variant), reference allele (CMS), alternative allele (MF) and annotation (ID) are reported (bold indicates a variant located within the exon).

CMS mtDNA Position	Type	Ref	Alt	ID	CMS mtDNA Position	Type	Ref	Alt	ID
4407^4408	Ins	-	T		210132	SNV	A	G	***atp6***
4413^4414	Ins	-	T		210134..210137	MNV	ACCC	GGAA	***atp6***
9932	Del	A	-		210140..210141	MNV	CC	TT	***atp6***
9937	Del	A	-		210143	SNV	A	G	***atp6***
17054	SNV	T	A		210145	SNV	C	T	***atp6***
17779	SNV	A	C		210148..210150	MNV	CGA	TAG	***atp6***
19036	Del	T	-		210152..210153	MNV	TA	GG	***atp6***
23779..23780	Del	GG	-	*nad4* (intron)	210155	SNV	T	G	***atp6***
26757^26758	Ins	-	G	*nad4* (intron)	210157..210158	MNV	CT	AG	***atp6***
29433	Del	G	-	*nad4* (intron)	210160..210163	MNV	ATAT	CCCC	***atp6***
38972^38973	Ins	-	A		210168..210171	MNV	GCTT	ATCC	***atp6***
85033	Del	A	-		210176	SNV	T	C	***atp6***
97184	Del	A	-		210179	SNV	T	A	***atp6***
97194	Del	C	-		210193..210194	MNV	AA	CC	***atp6***
103048	Del	T	-		210218	SNV	G	T	***atp6***
106177^106178	Ins	-	T		210225	SNV	G	C	***atp6***
109983	Del	G	-		210276	SNV	G	A	***atp6***
110419	SNV	T	G		210291	SNV	T	C	***atp6***
175249	SNV	T	C	***rps3***	210293	SNV	G	A	***atp6***
175458	SNV	C	G	***rps3***	210296	SNV	T	G	***atp6***
176686	SNV	G	A	***rps3***	210374	SNV	C	T	***atp6***
181657	Del	T	-	*cox2* (intron)	212667	Del	T	-	
210097..210102	Del	CCGCTC	-	***atp6***	213187	SNV	A	G	
210103..210105	MNV	CCA	TTT	***atp6***	213216	SNV	A	C	
210111	SNV	C	T	***atp6***	238616^238617	Ins	-	C	
210113	SNV	G	A	***atp6***	238643^238644	Ins	-	G	
210117	SNV	A	G	***atp6***	240379^240380	Ins	-	TTTATTAT	orf43
210117^210118	Ins	-	AG	***atp6***	247260	Del	C	-	
210122	SNV	A	G	***atp6***	248437	Del	A	-	
210124..210126	MNV	TAA	GTG	***atp6***	293347	Del	T	-	*nad2* (intron)
210129^210130	Ins	-	CTT	***atp6***	295317	Del	T	-	*nad2* (intron)
210130	SNV	A	T	***atp6***					

## Supplementary Materials

Supplementary materials can be found at https://www.mdpi.com/1422-0067/21/13/4664/s1.
