# Supplementary material for: The Mitochondrial Genome Assembly of Fennel (Foeniculum vulgare) Reveals Two Different atp6 Gene Sequences in Cytoplasmic Male Sterile Accessions
Source: Int J Mol Sci. 2020 Jun 30;21(13):4664. doi: 10.3390/ijms21134664 (PMC7370444; doi:10.3390/ijms21134664)
Supplement: Supplementary file 1 [file ijms-21-04664-s001.zip › Supplementary Figures 1 - 4.pdf]

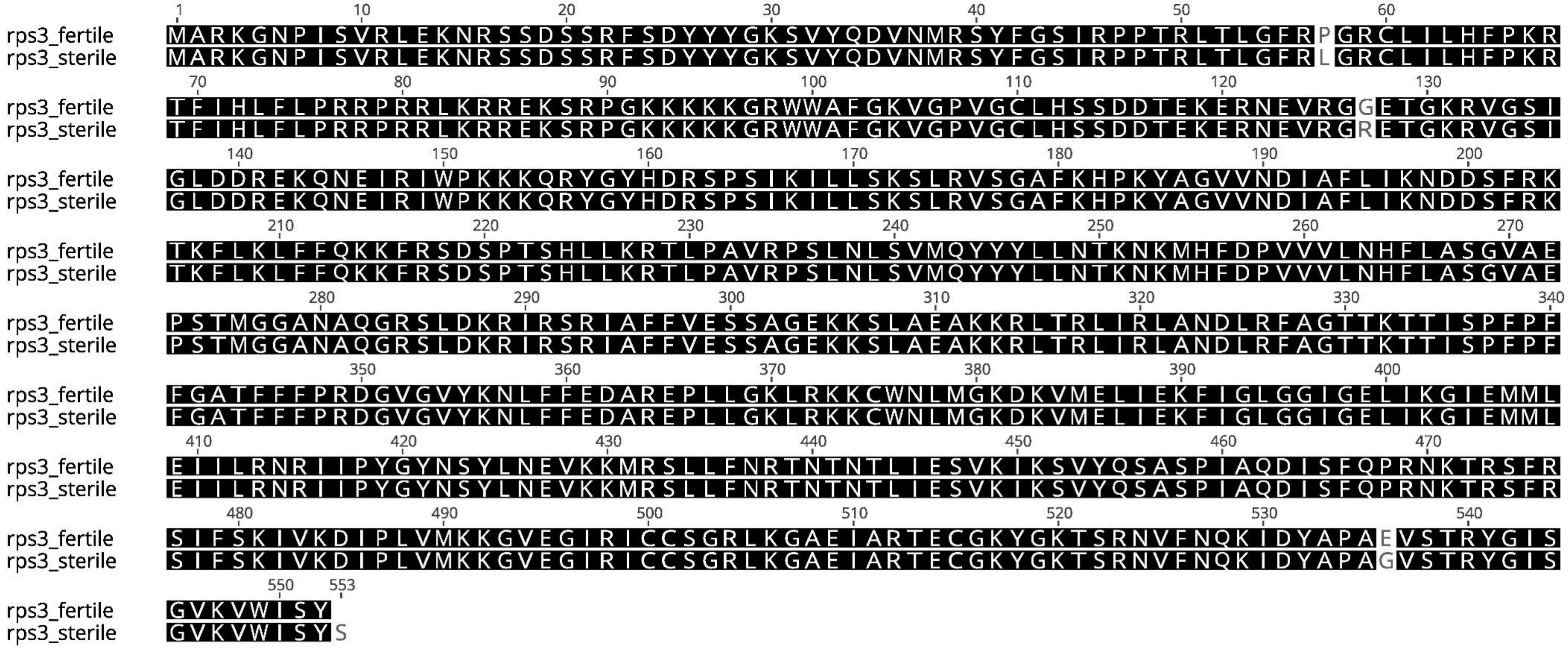

Figure S1 Amino acid alignment of ribosomal protein small subunits 3 (rps3) retrieved from the mtDNA of male fertile (MF, rps3 fertile) and cytoplasmic male sterile (CMS, rps3 sterile) accessions of *Foeniculum vulgare*

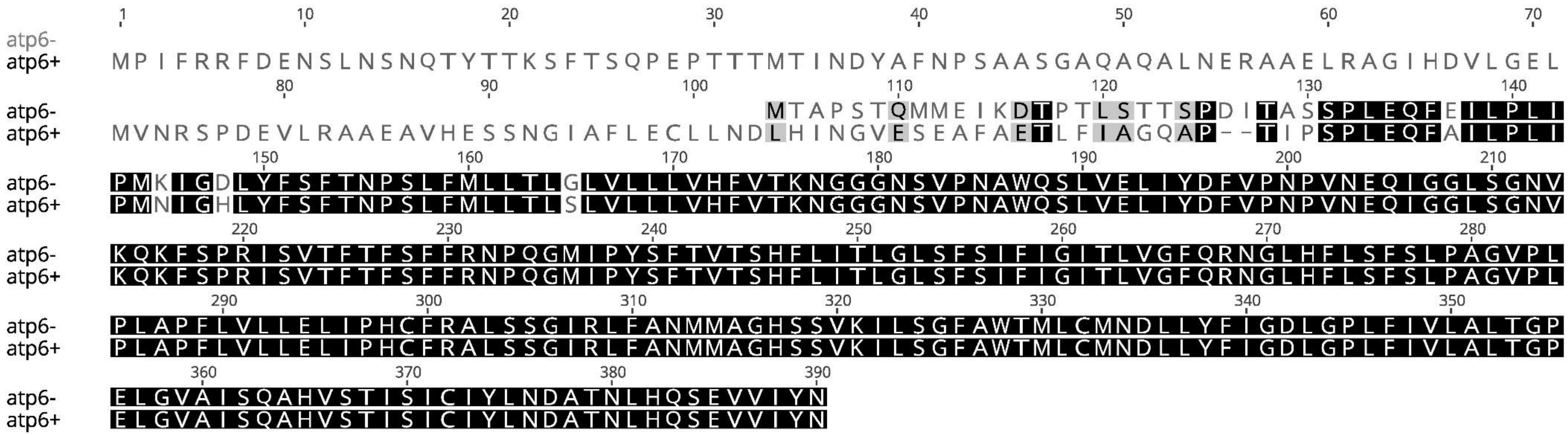

Figure S2 Amino acid alignment of atp6 retrieved from the mtDNA of male fertile (MF, atp6^{+}) and cytoplasmic male sterile (CMS, atp6^{-}) accessions of *Foeniculum vulgare*

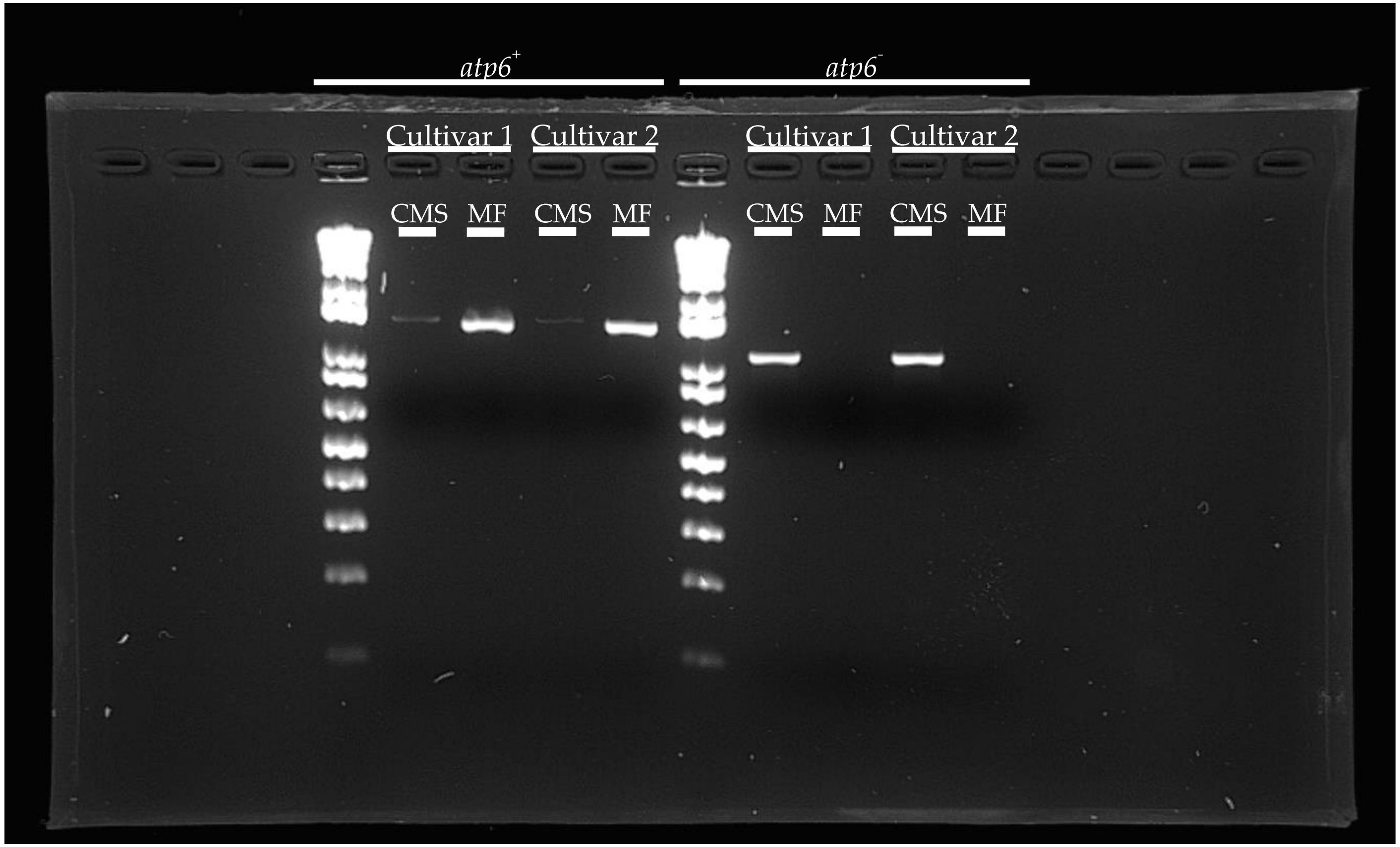

Figure S3. PCR amplification of two cytoplasmic male sterile individuals (CMS) from as many commercial lines and their two isogenic fertile maintainers (MF). In details, for the analysis of the wt copy of the *atp6* gene (*atp6*$^{+}$**)**, the following primers were used: F1: ATCGACCTGAACAACATATACGGA R1: GCTGGCGATTTCCGACAAGT. For the amplification of the mutated form (*atp6*$^{-}$), F2: TCACTGAGCACTGTCTG and R2: CCTAGAGTCTTTCGATACTATA were employed.

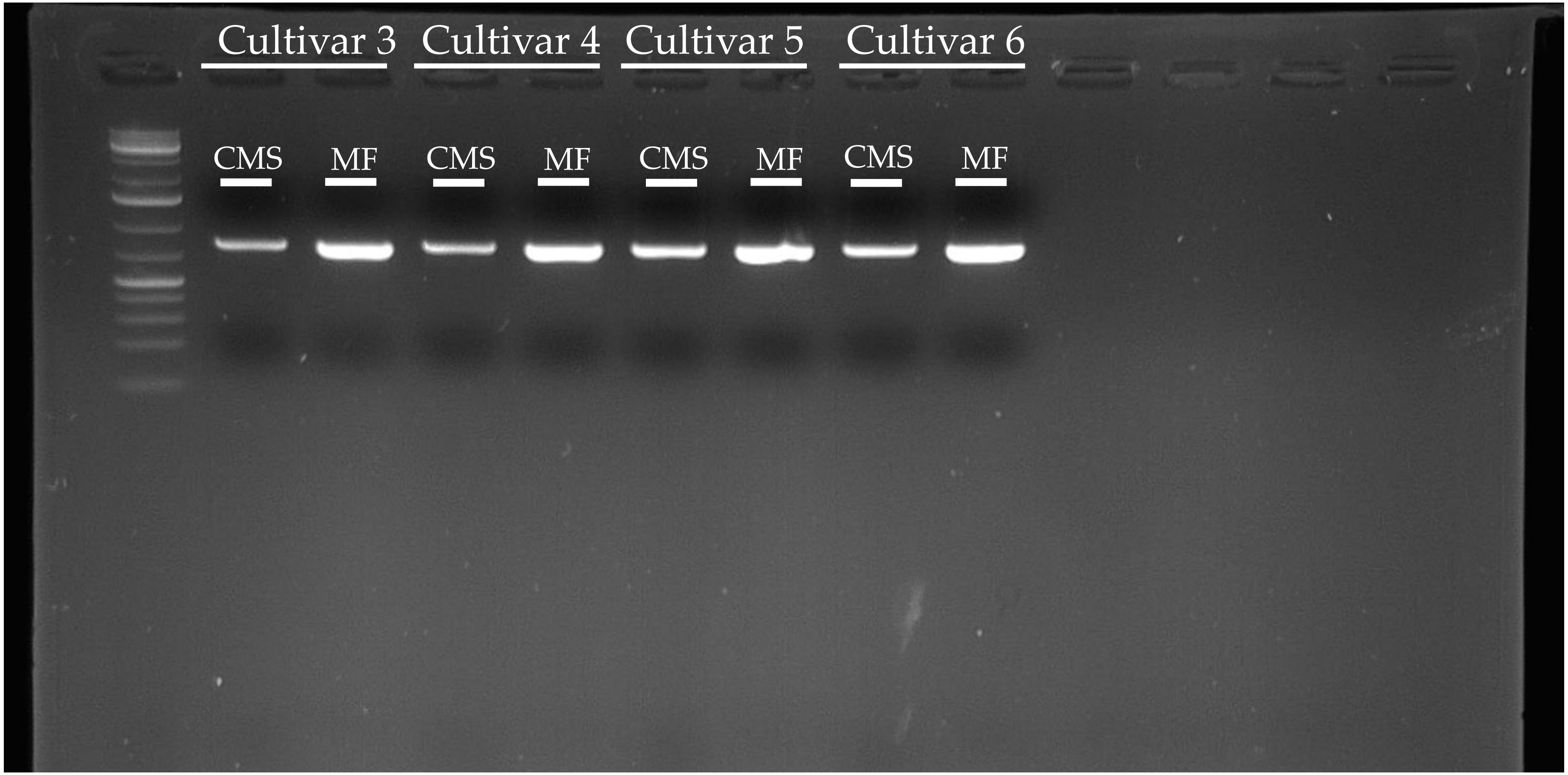

Figure S4. Additional PCR amplifications of the *atp6⁺* gene performed on four cytoplasmic male sterile individuals (CMS) from as many commercial lines (different from those ones used in the preliminary amplifications shown in Figure S3) and their isogenic fertile maintainers (MF).
